# Renal extramedullary hematopoiesis as an epiphenomenon of bone marrow dysfunction in a patient with primary myelofibrosis: A rare case report

**DOI:** 10.1016/j.radcr.2024.02.083

**Published:** 2024-03-21

**Authors:** Corrado Ini’, Pietro Valerio Foti, Andrea Duminuco, Renato Farina, Mariangela Clemenza, Francesco Tiralongo, Emanuele David, Stefano Palmucci, Giuseppe Alberto Palumbo, Antonio Basile

**Affiliations:** a Department of Medical Surgical Sciences and Advanced Technologies “G.F. Ingrassia”, University of Catania —Radiology I Unit, University Hospital Policlinico “G. Rodolico-San Marco”, Via Santa Sofia 78, 95123 Catania, Italy; b NANOMED-Research Centre for Nanomedicine and Pharmaceutical Nanotechnology, University of Catania, 95125 Catania, Italy; c Centro di Ricerca Multidisciplinare “Chirurgia delle Sindromi Malformative Complesse della Transizione e dell'Età Adulta” (ChiSMaCoTA), Department of Medical Surgical Sciences and Advanced Technologies “G.F. Ingrassia”, University of Catania, 95123 Catania, Italy; d Department of Medical Surgical Sciences and Advanced Technologies “G.F. Ingrassia”, University of Catania, 95123 Catania, Italy

**Keywords:** Hematopoiesis (G04.152.825, G09.188.343), Hematopoiesis, Extramedullary (G04.152.825.463, G09.188.343.463), Hematology (H02.403.429.445), Primary myelofibrosis (C15.378.190.636.765)

## Abstract

Extramedullary hematopoiesis represents a clinical compensatory condition characterized by the growth of hematopoietic tissue outside the bone marrow. It can mainly occur in patient with myeloproliferative disorders where alteration or neoplastic invasion of the bone marrow causes ineffective production of blood cells with the recruitment of progenitrix blood cells in non-hematopoietic organs, including kidneys. Renal extramedullary hematopoiesis is a rare condition manifesting as parenchymal or perirenal soft tissue masses with different patterns mimicking neoplasms, infectious or vascular diseases. We describe a unique case of a patient affected by primary myelofibrosis underwent ultrasound and magnetic resonance examinations showing bilateral perirenal alterations to be related to hemopoietic tissue. We also focused on the pathophysiology of this condition with imaging correlation. The case we present emphasises the importance of recognising the main radiological features of renal extramedullary hematopoiesis. MR examination should become part of the diagnostic pathway of the patient with primary myelofibrosis.

## Introduction

Extramedullary hematopoiesis (EMH) represents a medical condition in which hematopoietic tissue grows outside the bone marrow and involves organs with reticuloendothelial system cells, in particular spleen, liver, lymph nodes and, less commonly, lungs, brain, gastrointestinal tract, adrenal glands, and kidneys [1]. EMH mainly occurs in patients with myeloproliferative neoplasms (MPN) such as myelofibrosis, polycythemia vera or acute leukaemia, in which normal haematopoiesis fails due to alteration or neoplastic invasion of bone marrow leading to ineffective production of blood cells [2]. These conditions could cause morphological and tissue composition alterations in various organs, which are normally not involved in the production of hematopoietic cells after birth.

Myelofibrosis (MF) is a rare, chronic, malignant disease, primary (PMF) or secondary to other MPN (polycythemia vera and essential thrombocythemia), with extensive growth of fibrous tissue in the bone marrow. Bone marrow fibrosis impairs hematopoietic function which leads to extramedullary hematopoiesis in different anatomical locations, manifesting with hepatosplenomegaly, diffuse osteosclerosis, paravertebral soft tissue masses, or renal mass-like lesions. The kidney represents an uncommon site of EMH and typically appears as parenchymal and perirenal soft tissue masses, with infiltrating or mass-effect pattern [3].

Recognizing imaging features suggestive of EMH is essential since these alterations share certain features with neoplastic lesions. Radiological findings of EMH are manifold and heterogeneous and can be identified by ultrasound (US), computed tomography (CT), or magnetic resonance (MR) imaging examinations. MR allows the study of tissue composition identifying changes induced by EMH and it can assess signal intensity alteration in organs even before morphological changes [4].

We present a rare case of EMH involving both kidneys with intrapelvic soft tissues infiltrating masses in a patient with PMF, focusing on the imaging findings at MR and US examination.

## Case report

A 78-year-old male was referred to our hospital with severe anemia (Hb 7.6 g/dL), thrombocytosis (PLT 720,000/µL), and leukocytosis (WBC 20,500/µL). Considerable splenomegaly (longitudinal diameter of 16 cm) was detected at physical and ultrasound examination. Suspecting a chronic myeloproliferative disease, a bone marrow evaluation was performed showing 90% marrow cellularity, with hyperplasia of the granulocytic and megakaryocytic lines, <5% blast cells, and grade 1 fibrosis. Cytogenetic examination showed karyotype alterations (46, XY, del (20) (q12) in 19 out of 20 metaphases), and molecular biology examination revealed the presence of the *JAK2*V617F mutation. Based on these findings, a diagnosis of PMF was made, with an Intermediate-2 IPSS score (a total of 2 points for hemoglobin below 10 g/dL and age above 65 years, with an expected median overall survival -mOS- of 4.0 years) [5]. No other constitutional symptoms were reported. The patient started antiaggregant therapy (acetylsalicylic acid, 100 mg daily) and supportive recombinant erythropoietin (RHuEPO), 40,000 UI weekly, without achieving complete transfusion independence. Because of a rapid progressive leukocytosis, reaching a WBC value higher than 20,000/mm^3^ and increased platelet count (above 900,000/µl), in a few months, hydroxyurea (HU, 1000 mg daily) was added, obtaining an adequate blood count control. After 4 years, HU was stopped because of loss of efficacy (hyperleukocytosis, WBC 85000/mm^3^), progressive spleen enlargement (longitudinal diameter 18 cm at US evaluation), and concurrent diagnosis of squamous cell carcinoma of the skin, a relatively common side effect during therapy with HU [6]. At the same time, the patient reported the onset of constitutional symptoms (MPN-10 score equal to 30, with nocturnal sweating, fatigue, and inactivity), with a High DIPSS prognostic score, with an expected mOS of 1.5 years [7], [8]. A bone marrow biopsy was repeated, showing 100% cellularity, supported by fibroblasts, reduced granulopoiesis and erythropoiesis, and grade 3 reticulin fibrosis with the absence of blast cells, suggesting MF progression.

**Fig. 1:**
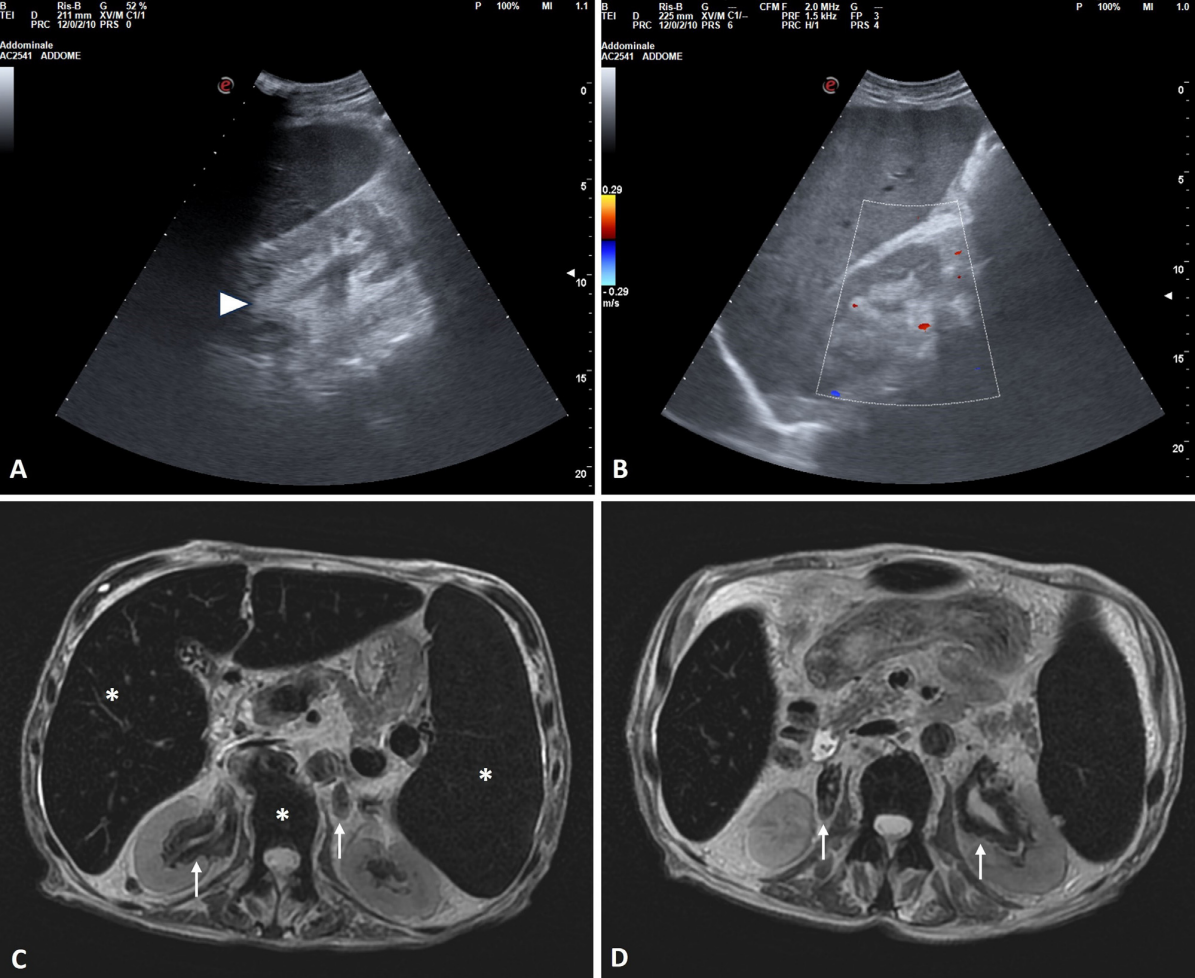
Imaging features of renal extramedullary hematopoiesis. (A, B) On ultrasound examination the right kidney shows hyperechoic tissue around the hilar and pelvic region (white arrowhead) extending to the proximal portion of ureter (A), with vessels visualization on color-doppler examination (B). (C, D) Axial T2-weighted turbo spin-echo images show parapyelic, peri-hilar and peri-ureteral solid bilateral renal lesions with infiltrative pattern and low signal intensity (white arrows). Signs of iron overload due to secondary hemochromatosis are evident in the liver, spleen and bone marrow with marked low signal intensity on T2-weighted turbo spin-echo sequences (white asterisks). Note splenomegaly.

**Fig. 2:**
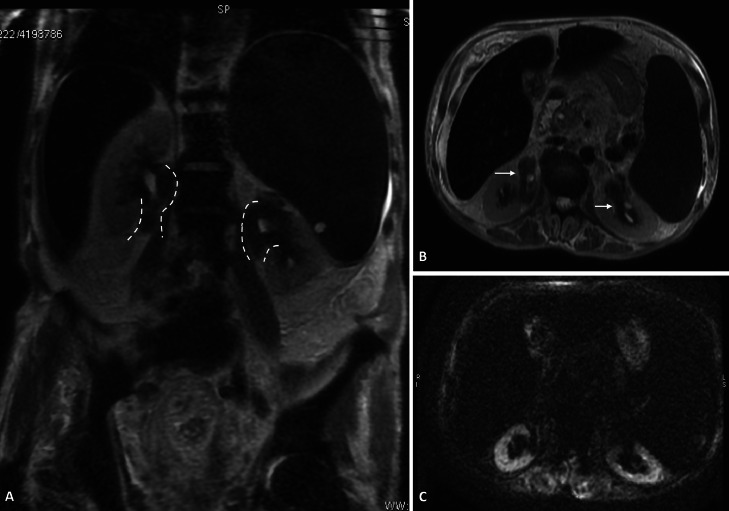
Radiologic follow-up in the same patient of Figure 1. The MR examination, performed 6 months later, confirmed the radiological pattern of bilateral renal lesions along the profile of the ureteropelvic junction (white dotted curved lines), showing low signal intensity on coronal (A) and axial (B) T2-weighted turbo spin-echo images (white arrows). (C) On axial DW image (b = 1000 s/mm^2^) the lesions do not exhibit restricted diffusion.

The patient was enrolled in a double-blind, randomized clinical trial based on the concurrent administration of ruxolitinib (JAK inhibitor) associated with navitoclax (BCL-2 inhibitor), or placebo [9]. Before starting treatment, scheduled US and MR examinations were performed. The patient underwent abdominal ultrasound examination in B-mode, assessing vascularity with color, power and duplex Doppler technique using an Esaote ultrasound device with a 3.5 Mhz convex probe. Ultrasonography showed splenomegaly (longitudinal diameter of 18 cm) and hyperechoic soft tissue masses around hilar and pelvic region of both kidneys extending to the proximal portion of ureters without generating a mass effect on adjacent anatomical structures and vessels (Figs. 1 A and B). MR examination of the abdomen was conducted with 1.5-T MRI scanner (Signa HDxT; GE Healthcare, Milwaukee, WI, USA - 57.2mT/m gradient strength and 120T/m/s slew rate) and confirmed splenomegaly (craniocaudal diameter: 20 cm; antero-posterior diameter: 18 cm, latero-lateral diameter: 12 cm; volume: 1952.66 cm^3^), abdominal effusion, signs of iron overload in liver and bone marrow and morphologic changes in both kidneys. In particular, kidneys were increased in size and presented parapyelic and peri-hilar solid renal lesions with marked low signal intensity on both T1- and T2-weighted sequences. These lesions showed infiltrative pattern in the peri-hilar space without significant mass-effect and extended to the proximal tract of the ureter (Figs. 1 C and D). The patient had no urinary tract symptoms and urinalysis was normal. Radiological features were suggestive of new neoplasm or EMH. Combined with the clinical data, our primary hypothesis was EMH, and we started a nephrological watch-and-wait approach. The follow-up MR examination, performed 6 months later, reported, unchanged, the radiological pattern of these lesions (Fig. 2), and the suspected diagnosis of EMH was confirmed.

## Discussion

Different hematologic disorders cause alteration in bone marrow with the recruitment of hemopoietic cells in other organs. The spleen and liver are the main sites of EMH, however, other sites may be recruited to produce hemopoietic cells. EMH in the kidney represents a rare condition and it can manifest as parenchymal, intrapelvic or perirenal lesions [10]. In our case, renal EMH occurred in a patient with PMF in absence of specific urinary tract symptoms. PMF is characterized by dysregulation of bone marrow microenvironment, with extensive growth of fibrous tissue, proliferation of megakaryocytes, and granulocytes leading to pancytopenia [11]. Therefore, EMH represents one of the pathological manifestations of PMF. Even if the reticuloendothelial system is the main site of EMH, other organs, like kidneys, contain hematopoietic precursors and can become a site of EMH. Hematopoietic and vascular endothelial cells are the first cells that differentiate following the mesoderm induction in the vertebrate embryo. In humans, hematopoietic stem cells (HSCs) originate from the aorta–gonad–mesonephros (AGM) region and then move to the fetal liver and finally to the bone marrow, which represents the definitive site of hematopoiesis in adults [12]. During the development of the yolk sac, hematopoiesis is closely related to the development of blood vessels through the presence of the hemangioblast, a common precursor for endothelial and hematopoietic cells. These findings suggest that vasculogenesis and hematopoiesis are part of the same process, with the formation of a blood vessel concurrent with the production in situ of blood cells from that vessel (hemo-vasculogenesis) [12]. The massive hematopoietic capacity of the embryo explains why extramedullary hematopoiesis could occur in any adult tissue under pathological conditions such as severe anemia or neoplastic bone marrow replacement like in PMF. In a recent study, Hwang et al. showed the existence of HSC-related markers in human fetal kidney cortex (SPI1 and RUNX1) and the presence of hematopoietic markers (RUNX1 and CD34) in kidney organoids derived from induced pluripotent stem cells (iPSCs) [13]. RUNX1 is essential in HSC generation, while SPI1, the main target downstream RUNX1, regulates hematopoiesis preventing uncontrolled HSC division. SPI1 and RUNX1 control different transcription factors sufficient to convert hemogenic endothelium into hematopoietic stem and progenitor cells. These data suggest the existence of a peculiar "kidney niche" that could appear transiently during the development of human embryo [13].

Renal EMH mainly manifests as intraparenchymal lesions with enlargement of the whole organ or with focal lesions or masses, creating problems of differential diagnosis with other medical conditions such as tumors, infectious or vascular diseases [10]. Perirenal involvement in EMH manifests as lobulated bilateral masses with uniform low attenuation on CT and may mimic renal lymphoproliferative disorders such as lymphoma. Pelvicalyceal or hilar involvement is the rarest type of renal EMH and it could manifest as soft tissue masses with bilateral infiltrative pattern, as in case we report. Hilar involvement may also cause obstructive renal failure with reduced renal function and elevated serum creatinine values. At the US, kidneys are enlarged and present iso-hyperechoic hilar or perirenal masses reflecting fat or myeloid content. At CT, renal EMH manifests as soft tissue mass with low fatty attenuation and enhancement after contrast media administration, reflecting the vascular nature of hemopoietic tissue. MRI is the best imaging modality to characterize tissue and internal content of focal lesions, thanks to its multiparametric nature. In general, MR signal intensity features of EMH are as follows: intermediate signal intensity on T1-weighted sequences, intermediate to high signal intensity on T2-weighted sequences and variable enhancement after paramagnetic contrast media administration. Nevertheless, EMH may also manifest as active or inactive lesions [4]. Active lesions present poor fat and iron infiltration but a higher component of myeloid cells, resulting in intermediate signal intensity on T1 sequences and high signal intensity on T2 sequences; these lesions show heterogeneous enhancement after contrast administration. Inactive lesions show extensive iron deposition determining low signal intensity on T1 and T2 sequences, due to susceptibility effects related to the superparamagnetic properties of the ions, which cause local distortion in the magnetic field [4]. In the case we report, at MR examination patient presents to our attention with bilateral infiltrative soft tissue masses in hilar regions of both kidneys, characterized by marked low signal intensity on T1 and T2-weighted sequences and, in particular, decreased signal intensity on T1-weighted in-phase sequences; this behaviour reflected iron overload due to repeated blood transfusions, configuring a condition of secondary hemochromatosis.

The diagnosis of renal EMH is often challenging since this condition presents overlapping features with other diseases. The knowledge of radiological characteristics and the use of MRI in typing the pathological tissue allows a correct non-invasive diagnosis to be made. The nuclear medicine could help in achieving the final diagnosis using technetium99m (99mTc) sulfur colloid scan, a radiopharmaceutical diagnostic agent employed in the evaluation of reticuloendothelial cells for the assessment of an ectopic source of hemopoietic precursors [14]. Unfortunately, not all hospitals perform this examination.

Less than 20 cases of renal EMH are described in the literature [15], and none of these have analyzed MR features of hilar renal EMH. Our case is quite unique because we detected hilar and pelvic-caliceal EMH in both kidneys with extensive iron deposition due to repeated blood transfusions. The final diagnosis was made through anamnesis and radiological findings at US and MR examination, since the biopsy was contraindicated in this patient.

## Conclusion

Renal EMH is a rare medical condition with heterogeneous radiological and clinical patterns. The involvement of the hilar region of both kidneys makes the differential diagnosis with other malignant conditions difficult. Biopsy provides a diagnosis of certainty but is not routinely performed due to the high risk of bleeding, since extramedullary hemopoietic tissue is characterized by a myeloid component, rich in blood vessels. Knowledge of different imaging patterns and the use of MR allows a correct diagnosis avoiding invasive diagnostic procedures. Finally, the role of ruxolitinib (or a different type of treatment in development for MF [16]) needs to be assessed in EMH, trying to identify the best approach in this setting of patients.

## Patient consent

Written informed consent for the publication of this case report was obtained from the patient.
